# Multiplicative interaction of functional inflammasome genetic variants in determining the risk of gout

**DOI:** 10.1186/s13075-015-0802-3

**Published:** 2015-10-13

**Authors:** Cushla McKinney, Lisa K. Stamp, Nicola Dalbeth, Ruth K. Topless, Richard O. Day, Diluk RW Kannangara, Kenneth M. Williams, Matthijs Janssen, Timothy L. Jansen, Leo A. Joosten, Timothy R. Radstake, Philip L. Riches, Anne-Kathrin Tausche, Frederic Lioté, Alexander So, Tony R. Merriman

**Affiliations:** Department of Biochemistry, University of Otago, Box 56, Dunedin, 9054 New Zealand; Department of Medicine, University of Otago, Christchurch, PO Box 4345, Christchurch, New Zealand; Department of Medicine, University of Auckland, Auckland, New Zealand; School of Medical Sciences, University of New South Wales, Sydney, Australia; Department of Clinical Pharmacology & Toxicology, St Vincent’s Hospital, Sydney, Australia; Department of Rheumatology, Rijnstate Hospital, Arnhem, The Netherlands; Department of IQ HealthCare, VieCuri Medical Centre, Venlo, The Netherlands; Scientific Institute of Quality in HealthCare, Radboud University Medical Centre, Nijmegen, The Netherlands; Department of Internal Medicine and Radboud Institute of Molecular Life Science, Radboud University Medical Center, Nijmegen, The Netherlands; Department of Rheumatology and Clinical Immunology, Laboratory of Translational Immunology, University Medical Centre Utrecht, PO Box 85500, 3508 GA Utrecht, The Netherlands; Department of Immunology, University Medical Centre Utrecht, PO Box 85500, 3508 GA Utrecht, The Netherlands; Rheumatic Diseases Unit, Institute of Genetics and Molecular Medicine, University of Edinburgh, Edinburgh, UK; Department of Rheumatology, University Clinic Carl-Gustav-Carus”, Dresden, Germany; INSERM, UMR-S 1132, Hospital Lariboisière, F-75010 Paris, France; University Paris Diderot (UFR de Médecine), Sorbonne Paris Cité, F-75205 Paris, France; DAL, Service of Rheumatology, Laboratory of Rheumatology, University of Lausanne, CHUV, Nestlé 05-5029, 1011 Lausanne, Switzerland

## Abstract

**Introduction:**

The acute gout flare results from a localised self-limiting innate immune response to monosodium urate (MSU) crystals deposited in joints in hyperuricaemic individuals. Activation of the caspase recruitment domain-containing protein 8 (CARD8) NOD-like receptor pyrin-containing 3 (NLRP3) inflammasome by MSU crystals and production of mature interleukin-1β (IL-1β) is central to acute gouty arthritis. However very little is known about genetic control of the innate immune response involved in acute gouty arthritis. Therefore our aim was to test functional single nucleotide polymorphism (SNP) variants in the toll-like receptor (TLR)-inflammasome-IL-1β axis for association with gout.

**Methods:**

1,494 gout cases of European and 863 gout cases of New Zealand (NZ) Polynesian (Māori and Pacific Island) ancestry were included. Gout was diagnosed by the 1977 ARA gout classification criteria. There were 1,030 Polynesian controls and 10,942 European controls including from the publicly-available Atherosclerosis Risk in Communities (ARIC) and Framingham Heart (FHS) studies. The ten SNPs were either genotyped by Sequenom MassArray or by Affymetrix SNP array or imputed in the ARIC and FHS datasets. Allelic association was done by logistic regression adjusting by age and sex with European and Polynesian data combined by meta-analysis. Sample sets were pooled for multiplicative interaction analysis, which was also adjusted by sample set.

**Results:**

Eleven SNPs were tested in the *TLR2*, *CD14*, *IL1B*, *CARD8*, *NLRP3*, *MYD88*, *P2RX7*, *DAPK1* and *TNXIP* genes. Nominally significant (*P* < 0.05) associations with gout were detected at *CARD8 rs2043211* (OR = 1.12, *P =* 0.007), *IL1B rs1143623* (OR = 1.10, *P* = 0.020) and *CD14 rs2569190* (OR = 1.08; *P* = 0.036)*.* There was significant multiplicative interaction between *CARD8* and *IL1B* (*P* = 0.005), with the *IL1B* risk genotype amplifying the risk effect of *CARD8.*

**Conclusion:**

There is evidence for association of gout with functional variants in *CARD8, IL1B* and *CD14*. The gout-associated allele of *IL1B* increases expression of IL-1β – the multiplicative interaction with *CARD8* would be consistent with a synergy of greater inflammasome activity (resulting from reduced CARD8) combined with higher levels of pre-IL-1β expression leading to increased production of mature IL-1β in gout.

**Electronic supplementary material:**

The online version of this article (doi:10.1186/s13075-015-0802-3) contains supplementary material, which is available to authorized users.

## Introduction

The immediate cause of gout is the deposition of monosodium urate (MSU) crystals in and around body tissues, particularly joints [1]. Initially, these deposits trigger a localised and self-limiting inflammatory response (acute gouty arthritis), which becomes increasingly frequent and severe, involving multiple joints and associated with fever. Monosodium urate crystals form under hyperuricaemic conditions when serum urate levels exceed the physiological saturation level (approximately 6.8 mg/dL; approximately 0.41 mM). The most significant biological cause of hyperuricaemia is relatively low renal clearance of uric acid [2, 3]. This is consistent with findings from genome-wide association studies in which 28 loci associated with serum urate levels have been identified, some of which are in genes involved in renal uric acid handling [4, 5]. Predictably most, but not all, of the 28 loci have been associated with gout [4, 6].

Although hyperuricaemia is a prerequisite for MSU formation, only a relatively small proportion of individuals with hyperuricaemia develop gout [7]. This indicates that beside genetic variants associated with urate metabolism and excretion, other factors contribute to the pathogenesis of gout. MSU crystals play an important role in activation of the innate immune system [8] and the recognition of gout as an auto-inflammatory disorder is consistent with the results of functional studies [9, 10]. Variations within genes of the innate immune system may therefore determine whether MSU crystals trigger an inflammatory reaction in susceptible individuals, leading to acute gout; while in others, no inflammation is elicited. Genetic variants that influence the activation and function of the NOD-like Receptor Pyrin containing 3 (NLRP3) inflammasome are candidate genes in this context [11]. The multi-protein inflammasome complex, comprising the NLRP3 polypeptide, ASC or PYCARD (apoptosis-associated speck-like protein containing a CARD) and caspase-1 [12] forms when monocytes and macrophages encounter damaged and pathogen-associated molecular pattern proteins (DAMPs and PAMP; e.g., bacterial lipopolysaccharide or MSU crystals) and leads to activation of caspase-1. Active caspase-1 processes the pro-interleukin (IL)-1β to the mature pro-inflammatory cytokine IL-1β that is then secreted [12].

CARD8 (also known as TUCAN or Cardinal) is a protein with a caspase-domain that interacts with caspase-1 and inhibits its activation [13] and also with a FIIND domain that binds to NLRP3 preventing its recruitment into the active inflammasome complex [14, 15]. Genetic associations between variants of CARD8 and autoimmune diseases have been previously reported (reviewed in [16]). The T allele of *CARD8 rs2043211* (C10X) has been associated with increased risk of gout in Chinese [17], and *rs2149356* in toll-like receptor 4 (TLR4), a receptor functionally implicated in MSU-stimulated inflammation [18], has also been associated with serum IL-1β levels and the risk of gout in Han Chinese [19]. However, using a haplotype tagging approach, there is no evidence of association between *NLRP3* and gout in Chinese [20].^1^

Our aim was to extend the findings from the Han Chinese population [17] and to test genetic variants influencing inflammasome function for association with gout in other population groups. Eleven functional variants were tested in eight genes involved in the MSU crystal-mediated activation of the NLRP3-inflammasome and production of mature IL-1β for association with gout in people of European and New Zealand (NZ) Polynesian (Māori and Pacific Island) ancestry. The prevalence of gout in the NZ Polynesian population is 6–8 % (compared to 3 % in NZ European), exhibiting the highest prevalence worldwide [21, 22].

## Methods

### Participants, ethics and consent

The study was carried out on sample sets comprising: a NZ Polynesian sample set consisting of 1,893 individuals of Samoan, Tongan, Niuean, Cook Island Māori and NZ Māori descent (Table 1; 863 cases and 1,030 controls); and a sample set consisting of 1,684 cases of European ancestry (957 recruited from NZ and Australia and 727 recruited from Europe (‘Eurogout’) [23]) and 882 NZ European controls. All people with gout met the 1977 preliminary American Rheumatism Association classification criteria for gout [24]. New Zealand gout cases were recruited from the Auckland and Christchurch regions of NZ, Australian gout cases from an outpatient clinic in Adelaide, Australian private practice rheumatologists and from a previously reported pharmacogenetic study [25]. European gout cases were recruited from outpatient clinics in Edinburgh, Lausanne, Dresden, Arnhem and Nijmegen. The NZ controls did not have any self-reported history of arthritis, were >17 years of age and were convenience sampled from the Auckland, Christchurch and Otago regions of NZ. The New Zealand Multi-Region Ethics Committee (MEC/105/10/130) and these institutional committees in Europe and Australia granted ethical approval: Research Ethics Committee, University of New South Wales; Ethikkommission, Technische Universität Dresden (EK 8012012); South East Scotland Research Ethics Committee (04/S1102/41); Commission Cantonale (VD) D'éthique de la Recherche sur l'être Humain, Université de Lausanne; Commissie Mensgebonden Onderzoek regio Arnhem Nijmegen. All subjects gave written and informed consent.

**Table 1:** Age, sex and serum urate details of studied sample sets

Sample set		Male,			Female,		
		number (%)	Average age^a^ (range)	Average serum urate mmol/L^b^ (range)	number (%)	Average age^a^ (range)	Average serum urate mmol/L^b^ (range)
Eastern Polynesian	Gout	365 (76.5)	37.8 (8–78)	0.47 (0.11–0.98)	112 (23.5)	51.0 (10–81)	0.45 (0.10–0.74)
	Control	268 (38.0)	42.5 (17–85)	0.41 (0.13–0.67)	437 (62.0)	46.5 (17–88)	0.34 (0.15–0.58)
Western Polynesian	Gout	341 (91.9)	35.1 (11–75)	0.49 (0.17–0.84)	30 (8.1)	43.8 (14–80)	0.49 (0.22–0.72)
	Control	175 (56.8)	38.3 (18–72)	0.42 (0.19–0.73)	133 (43.2)	41.2 (17–58)	0.35 (0.14–0.67)
Australasian	Gout	794 (82.7)	45.9 (5–92)	0.42 (0.11–0.80)	163 (17.3)	62.6 (20–90)	0.39 (0.13–0.74)
	Control	465 (53.0)	52.3 (18–87)	0.38 ( 0.18–0.77)	412 (47.0)	44.9 (17–95)	0.27 (0.13–0.59)
Eurogout	Gout	628 (86.4)	44.9 (16–83)	0.42 (0.09–0.92)	99 (13.6)	57.5 (30–92)	0.47 (0.13–0.88)
FHS	Control	1681 (53.7)	40.8 (19–69)	0.38 (0.13–0.67)	1450 (46.3)	43.9 (23–58)	0.26 (0.07–0.54)
ARIC	Control	3172 (45.4)	54.3 (44–66)	0.39 (0.08–0.71)	3817 (54.6)	55.6 (45–65)	0.31 (0.03–0.66)
All European	Gout	1422 (84.5)	45.5 (5–92)	0.42 0.09–0.92)	262 (15.5)	60.9 (20–92)	0.42 (0.13–0.88)
	Control	5318 (61.9)	50.0 (18–87)	0.38 (0.08–0.77)	5679 (38.1)	49.9 (17–95)	0.29 (0.03–0.66)

^a^Age at diagnosis for gout cases, at recruitment for controls. ^b^Serum urate levels at recruitment. Data presented for individuals for whom sex information was available

Control subjects of European ancestry, who had been genotyped genome-wide, were also included from the Atherosclerosis Risk in Communities (ARIC) study (n = 6,989) and the Framingham Heart Study (FHS; n = 3,131). Participants with self-reported gout were excluded. The sample sets were also screened to remove all but one representative of any closely related family grouping (full/half siblings and parent/child duos and trios). The ARIC study and FHS analyses (project #834) were approved by the relevant Database of Genotype and Phenotype (dbGaP; [26]) Data Access Committees.

### Genotyping and imputation

A Sequenom MassARRAY System was used for genotyping the 4,269 individuals without genome-wide genoytpe data available as previously described [27]. Publicly available genome-wide genotype data from ARIC and FHS were imputed as required to obtain genotypes from the eleven selected SNPs. Imputations were carried out by IMPUTE V2.2 software based on the combined 1000 Genomes V3 population reference set. Individuals and SNPs with a call rate of <0.98 were excluded from analysis, as were monomorphic markers and those with a minor allele frequency of <0.01, and a post-imputation quality threshold of 0.30 was used. Imputations were successful for all eleven SNPs and all imputed SNPs were in Hardy-Weinberg equilibrium (*P* >0.005) with the exception of *NLRP3 rs7512998* in ARIC (*P*_HWE_ = 1.3 × 10^-27^) - these data were excluded from analysis. Where necessary, imputed data were strand-adjusted to match the strand interrogated by the Sequenom data prior to analysis.

### Association testing

Allelic association analysis was carried out using R software and adjusting for age and sex. For Polynesian samples, a genetically estimated proportion of Polynesian ancestry, calculated as previously described [27], was included as an additional covariate. All odds ratios are reported relative to the minor allele present in the genotyped NZ European control sample set. Meta-analyses were carried out using METAL [28] with weighting based on standard error and log (odds ratio (OR)) as the effect variable. Significant association was declared if *P* was <0.05 in the European and Polynesian meta-analysis. No correction for multiple testing was applied because there is prior functional evidence for each variant tested.

Power curves are shown in Additional file 1. The Polynesian sample set was adequately powered to detect common effects (minor allele frequency (MAF) >0.1) of OR ≥1.4, whereas the European sample set was adequately powered to detect common effects of MAF >0.2 at OR >1.2 and stronger effects (OR >1.4) at lower MAF (>0.05).

### Interaction analysis

As reviewed by Cordell [29] there are a number of ways to test for interaction or a departure from additivity. We used Stata 13.1 software to carry out logistic regression analysis comparing the disease risk for heterozygosity and minor allele homozygosity for each locus individually and in combination. Previously we reported a large allele frequency difference at *ABCG2 rs2231142* and heterogeneity in association with gout between Eastern (EP) and Western (WP) Polynesian sample sets [30]. Similarly, there are differences in allele and genotype frequencies between the two groups (Additional file 2), and heterogeneity will be magnified in combined genotypes. Therefore, in the interaction analysis adjustment was made separately for EP and WP, along with European data. An interaction term was included and a *P* value <0.017 (Bonferroni-adjusted for number of interaction analyses performed) was considered to indicate significant multiplicative interaction between genetic variants.

## Results

Eleven functional genetic variants in *NLRP3, CARD8, IL1B, DAPK1, TXNIP, TLR2, P2XR7, MYD88* and *CD14* were selected from the literature (Table 2) and genotyped; genotype distributions are presented in Additional file 2. There was nominal allelic association (*P* <0.05) for three variants in the combined European and Polynesian analysis (Table 3) - *IL1B, CARD8* and *CD14* (OR = 1.10, 1.12 and 1.08, respectively). *CARD8 rs2043211* was also associated with gout in Europeans (OR = 1.11).

**Table 2:** Eleven genetic variants in *NLRP3, CARD8, DAPK1, TXNIP, TLR2, P2XR7, MYD88* and *CD14* were selected from the literature

SNP (gene)	Functional information	Association with auto-inflammatory phenotype
*rs10754558* (*NLRP3*) NLRP3 is a component of the NALP3 inflammasome.	Variant influences transcription (G > C) [46]	None
*rs35829419* (*NLRP3*)	Gain-of-function, with CARD8pC10X identified in arthritic patient with abnormally high IL-1 [47, 48]	Minor allele protective against celiac disease in a small study [49]
*rs7512998* (*NLRP3*)	None	Refer endnote 1 [20]
*rs2043211* (*CARD8*). CARD8 is a negative regulator of IL-1β secretion	C10X encodes a truncated protein that does not abrogate NFkB transcription [50]. Can be evaded by alternative splicing [51]. Identified as F102I in dbSNP	Interacts with NLRP3 rs35892419 in risk of Crohn’s disease [31]. Associated with disease severity in RA [50]
*rs1143623* (*IL1B*). IL-1β is a pro-inflammatory cytokine produced by activated NALP3 inflammasome	Promoter variant influences IL6 levels after fatty-acid rich meals [38] and IL-1β levels in small Crohn’s disease study [52]	G allele associated with protection from RA [53]
*rs4696480* (*TLR2*). Toll-like receptor involved in NALP3 inflammasome activation	Within binding site for the THP-1-derived nuclear protein, influences reporter expression [54]	None
*rs2569190* (*CD14*). CD14 is an adaptor molecule used by TLR2	*Rs2569190* T allele alters transcriptional activity [42, 43]	On meta-analysis is associated with Crohn’s disease [55] and asthma [56]
*rs6853* (*MYD88*). MYD88 is a transducer in the TLR signalling pathway	A allele creates potential miR-562b binding site [57]	None
*rs17525809* (*P2RX7*). Purine receptor involved in a pathway of NALP3 activation via amyloid A	Missense variant (Val > Ala), T allele reduces activity [58]	None
*rs4878104* (*DAPK1*). DAPK1 (death associated kinase) is involved in NALP3 assembly.	Exhibits allelic specific differences in expression [59]	None
*rs7212* (*TXNIP*). TXNIP is thioredoxin-interacting protein required for full inflammasome activation	Increased mRNA expression in smooth muscle cells with G allele [60]	None

*NALP3* NACHT, LRR and PYD domains containing protein 3, *CARD8* caspase recruitment domain-containing protein 8, RA rheumatoid arthritis, *TLR* toll-like receptor, *NLRP3* NOD-like Receptor Pyrin containing 3

**Table 3:** Analysis of associations between the minor alleles of the eleven variants and gout

	European				Polynesian				Meta-analysis	
Gene, SNP, minor allele	MAF case	MAF control	OR (95 % CI)	*P*	MAF case	MAF control	OR (95 % CI)	*P*	OR (StdErr)	*P*
*NLRP3, rs10754558,* G	0.408	0.418	0.95 (0.87–1.04)	0.25	0.459	0.434	1.07 (0.91–1.24)	0.42	0.96 (0.036)	0.44
*NLRP3, rs35829419,* A	0.046	0.047	0.88 (0.71–1.07)	0.20	0.004	0.012	0.58 (0.20–1.53)	0.29	0.87 (0.098)	0.15
*NLRP3, rs7512998,* C	0.170	0.160	1.09 (0.94–1.27)	0.25	0.033	0.045	1.03 (0.67–1.60)	0.88	1.08 (0.075)	0.29
*CARD8, rs2043211,* T	0.338	0.321	1.11 (1.01–1.22)	0.023	0.499	0.439	1.15 (0.98–1.35)	0.078	1.12 (0.042)	0.007
*IL1B, rs1143623,* G	0.278	0.266	1.09 (0.99–1.20)	0.066	0.532	0.481	1.14 (0.98–1.33)	0.098	1.10 (0.042)	0.020
*TLR2, rs4696480,* T	0.495	0.499	1.02 (0.93–1.11)	0.68	0.482	0.490	0.98 (0.84–1.15)	0.84	1.01 (0.036)	0.74
*CD14, rs2569190,* A	0.496	0.475	1.08 (0.99–1.18)	0.070	0.576	0.542	1.07 (0.92–1.26)	0.37	1.08 (0.036)	0.036
*MYD88, rs6853,* G	0.112	0.120	0.88 (0.77–1.00)	0.059	0.026	0.028	1.18 (0.71–1.98)	0.52	0.90 (0.068)	0.11
*P2RX7, rs17525809,* C	0.071	0.068	1.09 (0.92–1.29)	0.30	0.056	0.068	0.78 (0.56–1.08)	0.13	1.01 (0.080)	0.87
*DAPK1, rs4878104,* T	0.358	0.353	1.02 (0.93–1.11)	0.72	0.701	0.622	1.13 (0.96–1.34)	0.14	1.05 (0.044)	0.32
*TNXIP, rs7212,* G	0.050	0.041	1.27 (1.04–1.55)	0.020	0.191	0.175	1.00 (0.82–1.22)	0.98	1.13 (0.071)	0.091

*SNP* single nucleotide polymorphism, *MAF* minor allele frequency, *OR* odds ratio, *StdErr* standard error

Because of reported interactions between *NLRP3* and *CARD8* in other auto-inflammatory conditions [30, 31], and the biological interaction between the inflammasome and IL-1β we tested for pairwise multiplicative interaction between *NLRP3/CARD8*, *NLRP3/IL1B* and *CARD8/IL1B* (Table 4), using only *rs10754558* of *NLRP3* owing to the low MAF of *rs35829419* in both Europeans and Polynesians and the low MAF of *rs7512998* in Polynesians. There was evidence for interaction between *CARD8* and *IL1B* (*P* = 0.005, *P*_c_ = 0.015), driven by amplification of the risk conferred by the *CARD8 rs2043211* T allele in the presence of the *rs1143623 IL1B* minor allele homozygous (GG) genotype. Nominally significant interaction between *NLRP3/IL1B* (*P*_Nominal_ *=* 0.048) was also observed, although this was not significant after adjustment for multiple testing (*P*_c_ = 0.144).

**Table 4:** Interaction analysis: genotype combinations of *CARD8 rs2043211* and *NLRP3 rs10754558*, *CARD8 rs2043211* and *IL1B rs1143623,* and *NLRP3 rs10754558* and *IL1B rs1143623*

	Number (%)			
Combination	Cases	Controls	OR (95 % CI)	*P* ^a^
*rs10754558- rs2043211* (*NLRP3-CARD8*)				
CC/AA	318 (0.127)	1,862 (0.155)	1.00	
CC/AT	376 (0.150)	1,722 (0.144)	1.17 (0.96–1.42)	0.11
CC/TT	133 (0.053)	476 (0.040)	1.55 (1.18–2.04)	0.001
CG/AA	450 (0.180)	2,606 (0.217)	0.94 (0.78–1.13)	0.53
CG/AT	583 (0.233)	2,565 (0.214)	1.16 (0.97–1.38)	0.11
CG/TT	186 (0.074)	635 (0.053)	1.45 (1.11–1.81)	0.006
GG/AA	172 (0.069)	964 (0.080)	0.95 (0.75–1.20)	0.65
GG/AT	206 (0.082)	905 (0.076)	1.22 (0.97–1.54)	0.085
GG/TT	80 (0.032)	258 (0.022)	1.71 (1.23–2.39)	0.002
*rs1143623-rs2043211* (*IL1B-CARD8*)				
CC/AA	430 (0.174)	2,821 (0.236)	1.00	
CC/AT	488 (0.197)	2,740 (0.229)	1.13 (0.96–1.32)	0.16
CC/TT	136 (0.055)	644 (0.054)	1.26 (0.99–1.62)	0.066
CG/AA	390 (0.157)	2,182 (0.182)	1.10 (0.92–1.31)	0.28
CG/AT	495 (0.200)	1,979 (0.165)	1.48 (1.25–1.75)	4.3 × 10^−6^
CG/TT	162 (0.065)	575 (0.048)	1.74 (1.36–2.22)	8.7 × 10^−6^
GG/AA	112 (0.045)	425 (0.036)	1.33 (1.00–1.77)	0.049
GG/AT	166 (0.067)	465 (0.039)	1.61 (1.25–2.07)	2.0 × 10^−4^
GG/TT	100 (0.040)	147 (0.012)	3.77 (2.65–5.34)	<1.0 × 10^−6^
*rs10754558- rs1143623* (*NLRP3-IL1B*)				
CC/CC	352 (0.141)	2,147 (0.179)	1.00	
CC/CG	346 (0.139)	1,574 (0.131)	1.32 (1.09–1.60)	0.005
CC/GG	128 (0.051)	340 (0.028)	2.09 (1.57–2.78)	<1.0 × 10^−6^
CG/CC	497 (0.200)	2,963 (0.247)	1.00 (0.84–1.19)	1.00
CG/CG	529 (0.212)	2,312 (0.193)	1.30 (1.09–1.54)	0.004
CG/GG	184 (0.074)	524 (0.044)	1.43 (1.11–1.85)	0.005
GG/CC	209 (0.084)	1,099 (0.092)	1.22 (0.98–1.52)	0.072
GG/CG	179 (0.072)	851 (0.071)	1.14 (0.90–1.44)	0.272
GG/GG	66 (0.026)	173 (0.014)	1.70 (1.17–2.48)	0.006

^a^ *P*_Interaction_ was 0.91for *CARD8/NLRP3*, 0.005 for *CARD8/IL1B*, and 0.048 for *NLRP3/IL1B. OR* odds ratio

## Discussion

TLR signalling via the NLRP3 inflammasome has been implicated in gout susceptibility and pathology in vivo and in vitro [10]; for example, MSU uptake and IL-1β production by bone marrow-derived macrophages derived from TLR2, TLR4 or Myd88 knockout mice is significantly reduced, as is neutrophil influx, in response to subcutaneous injection of MSU in whole animals [18]. To further elucidate the role of the TLR-inflammasome-IL-1β cascade in gout pathogenesis, eleven candidate genetic variants that functionally impact on this pathway (reviewed in [10]) were tested for association with gout in a sample set of 2,357 cases, adequately powered to detect association with common variants having an effect size of odds ratio 1.4 or greater (Additional file 1). As discussed below, the nominal evidence for association between gout and *CD14, CARD8* and *IL1B*, and the multiplicative interaction between *CARD8* and *IL1B* in determining the risk of gout*,* support the considerable evidence that TLR-mediated activation of the inflammasome and subsequent release of active IL-1β is a central causal pathogenic pathway of gout [10, 32].

Variants *rs2043211* (*CARD8*), *rs1143623* (*IL1B*) and *rs2569190* (*CD14*), which were associated with gout, are functional variations in genes directly involved in the NLRP3 signaling pathway, and as such are likely to represent genuine disease-susceptibility loci. CARD8 is an adaptor protein that regulates IL-1β secretion by inhibiting NFKβ signaling (required for the expression of pro-IL-1β) and/or interacting with caspase 1 or NLRP3 to inhibit the generation of active IL-1β from inactive pro-IL-1β [13, 15]. The effect size of *CARD8* SNP *rs2043211* was consistent between the European and NZ Polynesian samples sets (OR = 1.11, *P* = 0.023 and OR = 1.15, *P* = 0.078, respectively; combined OR = 1.12, *P* = 0.007) and the Chinese sample set reported by Chen et al. (OR = 1.19, *P* = 0.08) [17]*.* Collectively our results and the study by Chen et al. [17] do suggest that the association of the minor allele of *rs2043211* with gout is not a false positive one. SNP *rs2043211* encodes a missense protein variation (C10X or F52I depending on transcript, [33]) with the minor allele increasing the risk of gout. Although the functional effect of this variation has not been specifically evaluated, the SNP is within an expression quantitative trait locus peak and carriage of the minor allele is associated with decreased CARD8 expression [34]. It has been inconsistently associated with other auto-inflammatory phenotypes, with some evidence for epistatic interaction with *NLRP3 rs35829419* in determining risk of inflammatory bowel disease and abdominal aortic aneurysms [31, 35]. However, we found no evidence of interaction between *NLRP3* and *CARD8* in determining risk of gout (Table 4) (although we did not specifically analyse *rs35829419*), and it has also been suggested that under certain conditions, ASC-dependent IL1-β production in response to MSU stimulation can occur in the absence of NLRP3 [36].

*IL1B* SNP *rs1143623* is within a promoter GATA transcription factor family binding site. Although the minor allele exhibits enhanced protein-binding [37] and decreased expression in vitro and in vivo, the effect of this variation seems to be influenced both by the wider promoter haplotype [38–40], and the identity of the stimulatory signal; the minor allele shows increased rather than decreased expression in vitro in response to TNF-α [40], and has also been associated with increased post-prandial triglyceride and IL6 (an effector of IL-1β) levels [41]. The minor allele is over-represented in gout cases compared to controls (OR = 1.10, *P* = 0.020). If this is replicated it would be consistent with an etiological role for increased *IL1B* expression in gout. There was evidence of multiplicative interaction with *CARD8 rs2043211* in which the *IL1B rs1143623* minor (risk) allele homozygous genotype appeared to amplify the effect of the minor allele of *rs2043211.* This would be consistent with a synergy of greater inflammasome activity (resulting from reduced CARD8) combined with higher levels of pre-IL-1β expression leading to increased production of mature IL-1β in gout.

The final variation nominally associated with gout was *CD14* SNP *rs2569190*. This SNP is in the 5’UTR of one of the two CD14 splice variants, with the minor allele (that increases risk of gout) increasing expression in monocytes by decreasing affinity for the inhibitory Sp3 transcription factor [42], enhancing the loading of RNA polymerase II [43] and is associated with increased soluble CD14 levels in healthy individuals [44, 45]. Membrane-bound CD14 forms functional complexes with TLR2 or TLR4 and leukocyte β2-integrins, which could mediate TLR dimerization and optimize the innate immune response to MSU crystals [10].

## Conclusions

In conclusion we provide evidence for association of gout with functional innate immune system variants in *CARD8, IL1B* and *CD14*, and multiplicative interaction between *IL1B* and *CARD8.* The findings involving *IL1B* are consistent with genetically determined levels of IL-1β being important in gout.

## Additional files

Additional file 1:
**Association power curves.** Figure presenting the power of the genetic association analysis. (PPTX 161 kb) Additional file 2:
**Genotype distributions.** Excel file with genotype distributions in the European and Polynesian sample sets for each of the single nucleotide polymorphisms (SNPs) studied. (XLSX 20 kb)

## Abbreviations

ARA: American Rheumatology Association
ARIC: Atherosclerosis Risk in Communities
ASC: apoptosis-associated speck-like protein containing a CARDD
CARD8: caspase recruitment domain-containing protein 8
DAMP: damage-associated molecular pattern
EP: Eastern Polynesian
FHS: Framingham Heart Study
IL: interleukin
MAF: minor allele frequency
MSU: monosodium urate
NALP3: NACHT, LRR and PYD domains-containing protein 3
NLRP3: NOD-like Receptor Pyrin containing 3
NOD: nucleotide-binding oligomerisation domain
NZ: New Zealand
OR: odds ratio
PAMP: pathogen-associated molecular pattern
UTR: untranslated region
RNA: ribonucleic acid
SNP: single nucleotide polymorphism
TLR: toll-like receptor
TNF: tumour necrosis factor
WP: Western Polynesian

## Footnotes

1 Note that the Meng et al paper [20] reported positive association with single nucleotide polymorphism (SNP) *rs7512998*, however the *P* value for this SNP had been incorrectly calculated - the true *P* value for *rs7512998* was 0.43 - as reported in a corrigendum [61].
