# Supplementary material for: Multiplicative interaction of functional inflammasome genetic variants in determining the risk of gout
Source: Arthritis Res Ther. 2015 Oct 13;17:288. doi: 10.1186/s13075-015-0802-3 (PMC4604627; doi:10.1186/s13075-015-0802-3)
Supplement: Additional file 1: — Association power curves. Figure presenting the power of the genetic association analysis. (PPTX 161 kb) [file 13075_2015_802_MOESM1_ESM.pptx]

## Slide 1
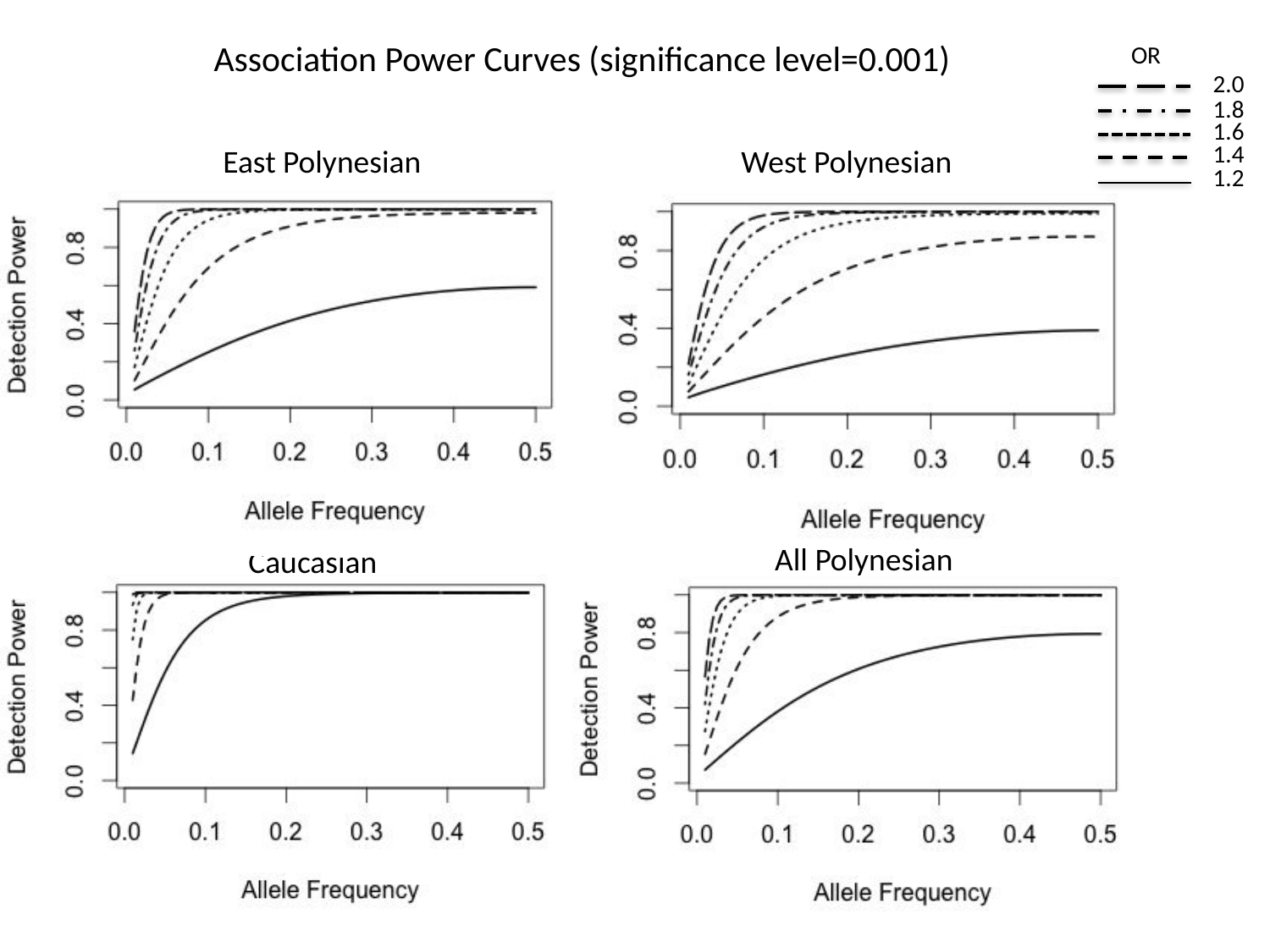

Association Power Curves (significance level=0.001)
OR
2.0
1.8
1.6
1.4
1.2
East Polynesian
West Polynesian
All Polynesian
Caucasian

## Slide 2
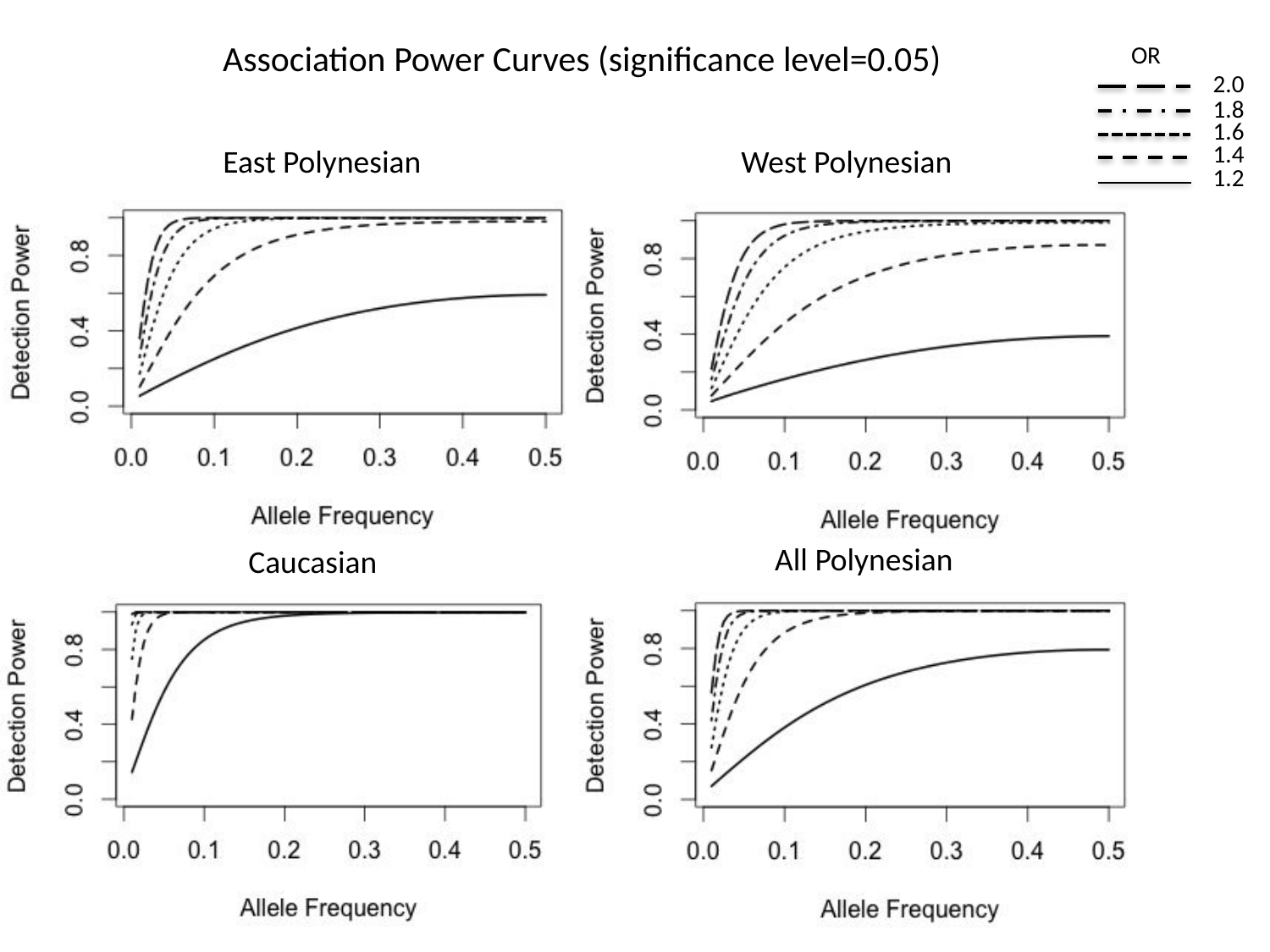

Association Power Curves (significance level=0.05)
OR
2.0
1.8
1.6
1.4
1.2
East Polynesian
West Polynesian
All Polynesian
Caucasian
